# Gender differences in cognitive reserve: An impact on progression in subjective cognitive decline?

**DOI:** 10.1002/dad2.70174

**Published:** 2025-08-26

**Authors:** Giulia Giacomucci, Valentina Moschini, Alice Ceccarelli, Sonia Padiglioni, Carmen Morinelli, Salvatore Mazzeo, Chiara Crucitti, Giulia Galdo, Filippo Emiliani, Silvia Bagnoli, Assunta Ingannato, Elisa Marcantelli, Benedetta Nacmias, Sandro Sorbi, Valentina Bessi

**Affiliations:** ^1^ Department of Neuroscience Psychology, Drug Research and Child Health University of Florence Florence Italy; ^2^ Research and Innovation Centre for Dementia‐CRIDEM AOU Careggi Florence Italy; ^3^ University of Florence Florence Italy; ^4^ Regional Referral Centre for Relational Criticalities ‐ Tuscany Region Florence Italy; ^5^ Vita‐Salute San Raffaele University Milan Italy; ^6^ IRCCS Policlinico San Donato San Donato Milanese Italy; ^7^ IRCCS Fondazione Don Carlo Gnocchi Florence Italy

**Keywords:** cognitive reserve, gender, mild cognitive impairment, subjective cognitive decline

## Abstract

**INTRODUCTION:**

This study investigated gender differences in cognitive reserve (CR) in subjective cognitive decline (SCD) and examined the impact of gender‐CR interaction on the risk of progression to mild cognitive impairment (MCI).

**METHODS:**

We enrolled 440 SCD patients and estimated CR using premorbid intelligence (*Test di Intelligenza Breve* [TIB]). To account for socio‐cultural differences, patients were stratified by birth cohort (pre‐/post‐1950). A Markov random‐field (MRF) model explored relationships between gender, CR, education, and age. Logistic regression assessed MCI progression risk.

**RESULTS:**

Women showed lower TIB scores than men (*p *< 0.001). The MRF model revealed an inverse connection between TIB and female gender, while no link was observed between TIB and generation. Progression to MCI was predicted by age at onset (*p *< 0.001), apolipoprotein E (*APOE)* status (*p *= 0.002), and TIB (*p *= 0.018), but not gender.

**DISCUSSION:**

Gender has an impact on CR, but not through socio‐economic variables. In turn, CR influenced the risk of MCI progression, whereas gender did not.

**Highlights:**

- Subjective cognitive decline (SCD) women presented lower cognitive reserve (CR) levels than men, despite similar education levels.
- Social‐cultural factors did not explain these gender differences in CR in SCD.
- The gender–CR interaction was not mediated by social–cultural factors.
- The risk of progression to mild cognitive impairment (MCI) was influenced by CR but not by gender.

## 1 INTRODUCTION

There is growing evidence identifying subjective cognitive decline (SCD) as a preclinical stage of Alzheimer's disease (AD).^1^ SCD has been defined as a self‐reported cognitive decline despite normal performance on standardized cognitive tests.^2^, ^3^ The National Institute of Aging‐Alzheimer's Association (NIA‐AA) included SCD as the earliest symptomatic expression of AD. Nevertheless, SCD is a heterogeneous entity, with several underlying causes, including mood disorders, sleep disturbances, and other medical or psychological factors.^2^, ^4^ Thus, till today SCD constitutes a heterogeneous group that need to be better characterized to identify those patients with the greatest risk of progression to mild cognitive impairment (MCI) and AD dementia.

**RESEARCH IN CONTEXT**

1. **Systematic review**: Cognitive reserve (CR) is a key resilience factor against cognitive decline in Alzheimer's Disease.
2. **Interpretation**: In individuals with subjective cognitive decline (SCD), women exhibit lower CR levels than men. This female disadvantage does not appear to be driven by socio‐cultural factors, suggesting an intrinsic link between gender and CR. While CR influences the risk of progression to mild cognitive impairment (MCI), gender itself does not play a direct role. This implies that other factors may modulate the risk in women, preventing their lower cognitive reserve from translating into a higher likelihood of progression.
3. **Future directions**: These findings highlight the complexity of the relationship between gender, CR, and cognitive decline, emphasizing the need for further research to uncover the underlying mechanisms.

One factor contributing to explain differences in individuals’ susceptibility to develop cognitive decline is cognitive reserve (CR). According to Stern's model, individuals with higher reserve can better tolerate a higher burden of AD neuropathology and maintain functioning longer, though they may experience faster decline once symptoms appear.^5^ While the role of CR in SCD is still under investigation, some studies suggested that high CR seems to act as a protective factor for progression from SCD to MCI.^6^ On the contrary, according to Stern's model, high CR resulted as a risk factor for progression from MCI to AD dementia.^6^ However, further longitudinal works would be needed to establish firmer evidence of CR protective effects in SCD.

Importantly, the relationship between CR and SCD appears complex and influenced by other factors, such as gender, as shown in other neurodegenerative diseases.^7^ Evidence suggests that women with SCD may have lower premorbid intelligence (a proxy of CR) than men with similar education levels,^8^ possibly due to biological or genetic differences, but also to historical social inequalities in access to education and cognitively stimulating occupations. These gender‐related disparities, especially across generations, may have lasting effects on CR development.^9^

In addition, it is not known whether the lower CR of SCD women increases the risk of progression to an “objective” cognitive decline compared to SCD men.

Therefore, to thoroughly investigate the complex role of gender in CR among individuals with SCD, it is necessary to adopt a methodological approach capable of modeling the interrelations between gender, CR proxies, and relevant demographic factors, including generational effects. Considering these premises, the aims of this study were: to deeply explore gender differences in CR (estimated as “premorbid intelligence”) in SCD, evaluating with the network analysis possible connections of gender with other variables which might explain this complex relationship; to investigate if the interaction between gender and CR may have a different impact on the risk of progression to MCI in women and men.

## 2 MATERIALS AND METHODS

### 2.1 Participants

This study was part of the ongoing longitudinal study titled “Predicting the Evolution of Subjective Cognitive Decline to Alzheimer's Disease With Machine Learning (PREVIEW)” (ClinicalTrials.gov Identifier: NCT05569083).^10^ We considered 440 patients referring to Center for Research and Innovation in Dementia (CRIDEM) of Careggi Hospital in Florence between January 1994 and November 2023. Inclusion criteria were: (1) complaining of cognitive decline with a duration of ≥6 months; (2) normal functioning on the activities of daily living and the instrumental activities of daily living scales^11^; (3) fulfilling SCD diagnostic criteria^3^; (4) unsatisfied criteria for dementia or MCI at baseline.^12^, ^13^ Exclusion criteria were history of head injury, current neurological and/or systemic disease, symptoms of psychosis, major depression, substance use disorder.

All participants underwent a comprehensive family and clinical history, general and neurological examination, extensive neuropsychological investigation described elsewhere.^10^ CR was estimated as premorbid intelligence, which was evaluated at baseline by the *Test di Intelligenza Breve* (TIB, i.e., Brief Intelligence Test),^6^, ^8^, ^14^, ^15^ an Italian version of the National Adult Reading Test (NART).^16^ The presence and severity of depressive symptoms was evaluated by means of the 22‐item Hamilton Depression Rating Scale (HDRS): scores ranges between 0 and 67, with higher scores indicating more severe depressive symptoms.^17^ The subjective perception of memory impairment (severity of SCD) was investigated using the Memory Assessment Clinics‐Questionnaire (MAC‐Q): scores ranges between 0 and 25, with higher scores indicating worsen complaints.^18^ According to year of born, patients were subdivided into two generation subgroups: pre‐1950 and post‐1950 generations. This classification by generation of birth was made as a proxy for social and cultural factors that might play a role in the construction of CR. Two hundred subjects underwent apolipoprotein E (*APOE)* genotyping.^19^ Positive family history was defined as one or more first‐degree relatives with documented cognitive decline. In all cases, there was a perfect correspondence between sex (the biological designation) and gender (the social construct). We defined as “insomnia” difficulties in falling asleep or waking up early or having frequent sleep interruptions.

Patients underwent clinical and neuropsychological follow‐up every 12 or 24 months. Progression from SCD to MCI was defined according to the NIA‐AA criteria.^13^ For longitudinal analyses, we included SCD patients who progressed to MCI (SCD‐p) or SCD patients who remained stable with a follow up time > 5 years (SCD‐s), according to Jessen et al. definition.^3^

The local ethics committee approved the protocol of the study. All participants gave written informed consent to participate in the study.

### 2.2 Statistical analysis

All statistical analyses were performed with Jamovi (Jamovi version 2.3), IBM SPSS Statistics Software Version 25 (SPSS Inc., Chicago, USA) and the computing environment R4.2.3 (R Foundation for Statistical Computing, Vienna, 2013). The distributions of variables were assessed using the Shapiro‐Wilk test. Patients’ groups were characterized using means and standard deviations (SD). All *p*‐values were two‐tailed and significance level for all analyses was set at α = 5%, corresponding to a threshold *p* of 0.05. Depending on the distribution of our data, we used *t*‐test or non‐parametric Mann–Whitney U tests for between‐groups, comparisons and Pearson's correlation coefficient or non‐parametric Spearman's ρ (rho) to evaluate correlations between groups’ numeric measures. We used chi‐squared test to compare categorical data. Differences among groups in continuous variables were assessed through one‐way analysis of variance (ANOVA) followed by Bonferroni post‐hoc test. Effect sizes was computed using η^2^ for the Mann‐Whitney U Test, and Cramer's V for categorical data.

To explore the relationship among features at baseline, we applied a network analysis based on graph theory. To create the networks (based on Markov random field [MRF] modeling), we used the the polychoric correlation method for the severity and distress data, using the R‐package qgraph^20^, ^21^ in combination with the graphical “least absolute shrinkage and selection operator” (glasso) algorithm with a tuning parameter of 0.25.^22^ Edge–edge and node–node comparisons were run using the “differenceTest” function within the bootnet package, without applying multiple comparison correction. We estimate three centrality indices: strength (quantifying how well a node is directly connected to other nodes), closeness (quantifying how well a node is indirectly connected to other nodes), and betweenness (quantifying how important a node is in the average path between two other node). We tested for significant differences between edges weights and centrality indices based on 1000 nonparametric bootstrap iterations. To estimate the stability of the order of the centrality indices, we used a case‐dropping bootstrap technique together with the correlation stability coefficient (Cs‐coefficient). To interpret centrality differences the CS‐coefficient should not be below 0.25.^23^

Finally, we constructed logistic regression models to predict the effect of demographic and clinical variables on the risk of progression from SCD to MCI (progression or not) and a multiple linear regression model to define which variables might influence the time of progression to MCI (in years).

## 3 RESULTS

### 3.1 Description of the sample, differences between genders and between generations

Demographic features of the whole sample and of gender subgroups are reported in Table 1. Women had lower TIB scores when compared to men (109.6 ± 6.9 vs. 114.9 ± 5.0, *p *< 0.001, d = 0.887). Men had lower HDRS and MAC‐Q scores than women (respectively, HDRS 26.4 ± 3.9 vs. 28.0 ± 4.9, *p *= 0.002, d = 0.358; MAC‐Q 25.0 ± 2.4 vs. 26.2 ± 3.3, *p *= 0.002, d = 0.425). No differences were found in years of education between women and men.

**TABLE 1:** Demographic data in the whole cohort and comparison between women and men.

Parameter	Whole cohort	Women	Men
	*n* = 440	*n* = 304	*n* = 136
Women	304 (69.1%)	–	–
Men	136 (30.9 %)		
Age at baseline in years	62.4 (± 9.2)	61.8 (± 9.1)	63.8 (± 9.3)
Age at onset in years	58.3 (± 9.9)	57.6 (± 9.8)	59.9 (± 10.1)
Disease duration in years	4.2 (± 3.8)	4.2 (± 3.9)	3.9 (± 3.6)
Family history of AD	56.6%	56.6%	56.6%
*APOE* ɛ4+	28.5%	24.3%	39.3%
Generation (born after 1950)	43.4%	46.0%	37.5%
Insomnia	54.6%	58.5%	46.1%
Menopause	–	89.1%	–
Age at menopause	–	50.0 (± 4.5)	–
Years of education	12.4 (± 4.3)	12.1 (± 4.4)	13.2 (± 4.1)
TIB	111.2 (± 6.8)	**109.6 (± 6.9)** ^*^	**114.9 (± 5.0)** ^*^
MMSE	27.9 (± 2.1)	27.9 (± 2.1)	28.1 (± 2.1)
HDRS	27.5 (± 4.7)	**28.0 (± 4.9)** ^+^	**26.4 (± 3.9)** ^+^
MAC‐Q	25.9 (± 3.1)	**26.2 (± 3.3)** °	**25.0 (± 2.4)** °

Values quoted in table are mean (± SD), or percentages. Statistically significantly different values between males and females are reported as **bold characters**. Statistical significancy after Bonferroni correction: *p *= 0.004.

Abbreviations: APOE, apolipoprotein E; TIB, *Test di intelligenza breve*; HDRS, Hamilton Depression Rating Scale; MAC‐Q, Memory Assessment Clinics‐Questionnaire.

^*^ *p *< 0.001, Cohen's d = 0.887.

^+^ *p *= 0.002, Cohen's d = 0.358.

° *p *= 0.002, Cohen's d = 0.425.

Age at baseline and age at onset were lower in patients of post‐1950 generation than in those of pre‐1950 one (age at baseline 56.0 ± 7.8 vs. 67.3 ± 6.8, *p *< 0.001, d = 1.536; age at onset 52.0 ± 9.2 vs. 63.1 ± 7.6, *p *< 0.001, d = 1.314). Moreover, patients of post‐1950 generation had higher education than those of pre‐1950 one (13.5 ± 43.6 vs. 11.6 ± 4.6, *p *< 0.001, d = 0.455) and higher HDRS scores (28.0 ± 4.9 vs. 26.4 ± 3.9, *p *= 0.002, d = 0.372). No differences in TIB scores were detected between the two generations (eTable 1).

Dividing women and men according to generation (Table 2), age at baseline and age at onset were lower in women of post‐1950 generation than in those of pre‐1950 one (respectively, age at baseline 55.7 ± 7.6 vs. 67.0 ± 6.6, *p *< 0.001, d = 1.593; age at onset 51.3 ± 8.6 vs. 62.9 ± 7.4, *p *< 0.001, d = 1.447). Furthermore, women of post‐1950 generation had higher education those women of pre‐1950 one (13.6 ± 3.4 vs. 10.8 ± 4.7, *p *< 0.001, d = 0.687). No differences in TIB score between generations were detected. In men, age at baseline and age at onset were lower in men of post‐1950 generation than in those of pre‐1950 one (respectively, age at baseline 57.0 ± 8.5 vs. 67.9 ± 7.2, *p *< 0.001, d = 1.384; age at onset 53.9 ± 1.04 vs. 63.4 ± 8.1, *p *< 0.001, d = 1.016). Post‐1950 generation men presented lower TIB scores than those of pre‐1950 one (112.8 ± 5.5 vs. 116.2 ± 4.2, *p *= 0.001, d = 0.500). No difference in educational level was found between generations.

**TABLE 2:** Demographic data in women and men classified according to generation.

	Pre‐1950 generation	Post‐1950 generation
Women	*n* = 164	*n* = 140
Age at baseline in years	**67.0 (± 6.6)** *	**55.7 (± 7.6)** *
Age at onset in years	**62.9 (± 7.4)** ^	**51.3 (± 8.6)** ^
Disease duration in years	4.1 (± 3.5)	4.4 (± 4.4)
Family history of AD	54.3%	59.4%
*APOE* ɛ4+	25%	23.3%
Insomnia	60.9%	55.4%
Menopause	**99.3%** °	**74.8%** °
Age at menopause	50.2 (± 4.7)	49.6 (± 4.2)
Years of education	**10.8 (± 4.7)** ^§^	**13.6 (± 3.4)** ^§^
TIB	109.4 (± 7.2)	109.9 (± 6.4)
MMSE	28.1 (± 1.9)	27.8 (± 2.2)
HDRS	27.4 (± 4.4)	28.9 (± 5.3)
MAC‐Q	26.2 (± 3.2)	26.3 (± 3.4)

	Pre‐1950 generation	Post‐1950 generation
Men	*n* = 85	*n* = 51
Age at baseline in years	**67.9 (± 7.2)** ^+^	**57.0 (± 8.5)** ^+^
Age at onset in years	**63.4 (± 8.1)** ^#^	**53.9 (± 10.4)** ^#^
Disease duration in years	**4.5 (± 3.5)** ^&^	**3.1 (± 3.6)** ^&^
Family history of AD	50.0%	67.3%
*APOE* ɛ4+	25%	23.3%
Insomnia	48.1%	42.5%
Years of education	13.2 (± 4.0)	13.2 (± 4.2)
TIB	**116.2 (± 4.2)** ^†^	**112.8 (± 5.5)** ^†^
MMSE	28.1 (± 2.0)	28.1 (± 2.2)
HDRS	25.7 (± 3.4)	27.7 (± 4.6)
MAC‐Q	25.4 (± 2.9)	24.5 (± 1.5)

Values quoted in table are mean (± SD), or percentages. Statistically significantly different values are reported as **bold characters**. Statistical significancy after Bonferroni correction: *p *= 0.004.

Abbreviations: AD, Alzheimer's disease; APOE, apolipoprotein E; HDRS, Hamilton Depression Rating Scale; MAC‐Q, Memory Assessment Clinics‐Questionnaire; MMSE, Mini‐Mental State Examination; TIB, *Test di intelligenza breve*.

* *p *< 0.001, Cohen's d = 1.593.

^ *p *< 0.001, Cohen's d = 1.447.

° χ^2 ^= 39.1, *p *< 0.001, Cramer's V 0.39.

^§^ *p *< 0.001, Cohen's d = 0.687.

^+^ *p *< 0.001, Cohen's d = 1.384.

^#^ *p *< 0.001, Cohen's d = 1.016.

^&^ *p *= 0.001, Cohen's d = 0.396.

^†^ *p *= 0.001, Cohen's d = 0.500.

### 3.2 Relationship among gender, CR, generation, and age at onset

TIB was directly correlated with years of education in the whole sample (ρ 0.681, *p *< 0.001) and in women and men separately (women ρ 0.702, *p *< 0.001; men ρ 0.742, *p *< 0.001). Similarly, TIB was directly correlated with education in both pre‐1950 and post‐1950 generations (pre‐1950 ρ 0.675, *p *< 0.001; post‐1950 ρ 0.766, *p *< 0.001). In the whole sample, TIB was directly correlated with age at baseline (ρ 0.255, *p = *0.002) and with age at onset (ρ 0.222, *p <* 0.001). On the other hand, years of education were inversely correlated with age at baseline (ρ ‐0.144, *p *= 0.002) and with age at onset (ρ ‐0.159, *p *= 0.001). In women, TIB was directly correlated and with age at baseline (ρ 0.193, *p *= 0.002), while years of education were inversely correlated with both age at baseline (ρ ‐0.263, *p *< 0.001) and age at onset (ρ ‐0.290, *p *< 0.001). In men, TIB was directly correlated with age at baseline (ρ 0.438, *p *< 0.001) and age at onset (ρ 0.438, *p *< 0.001), while no correlations between years of education, age at baseline and age at onset were found.

### 3.3 Network analysis

To better understand the relationship between gender, TIB, education, generation, and other demographic variables, we constructed a pairwise MRF model, considering the following as nodes: age at onset, gender, TIB, years of education, generation, symptoms duration, HDRS, MAC‐Q, insomnia. Robustness analyses of the edges and the centrality indices showed the following CS‐coefficients: 0.673 for edges, 0.439 for strength, 0.127 for closeness, 0.05 for betweenness. The network was robust regarding edges and strengths but was not acceptably robust regarding closeness and betweenness (Supplementary Figure).

**FIGURE 1:**
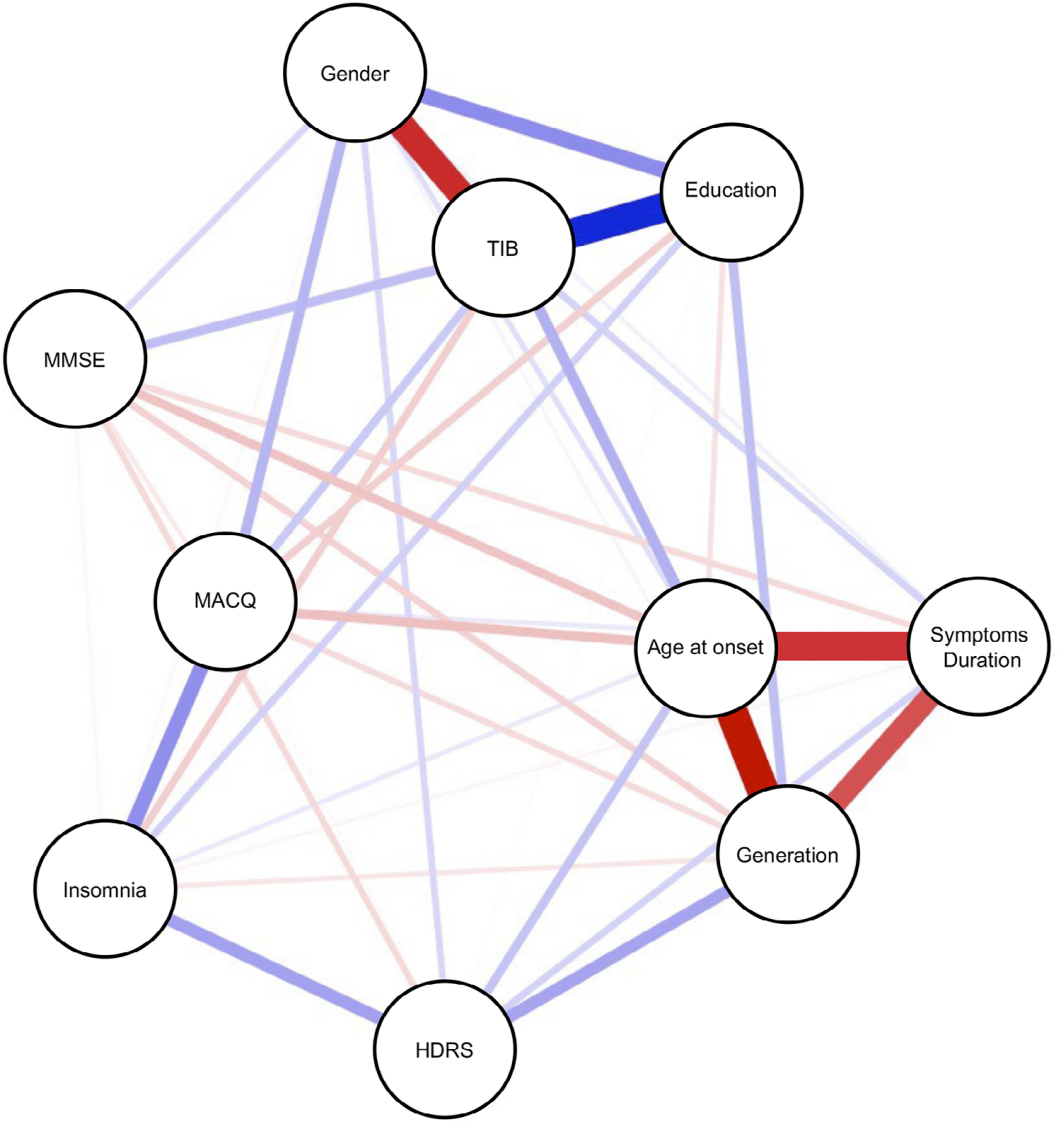
Estimated network. Thickness of the line between features (edges) indicates weight of edges. Blue = direct connection. Red = inverse connection. Categorical variables were coded as follows: Gender (0 = male, 1 = female); Generation (0 = pre‐1950, 1 = post‐1950). “Maximum 0.77″ refers to the the highest edge weight in the network.

Figure 1 represents the estimated network. We focused on the connection between female gender, TIB, education, generation, and age at onset. TIB and years of education were strongly directly connected, with a weight significantly higher than all the other edges. The estimated network showed an inverse connection between post‐1950 generation and age at onset, with the highest weight than all the other edges. Female gender and TIB scores showed a strong inverse connection too, with a weight significantly stronger than all the other edges, except for the connection between post‐1950 generation and age at onset, age at onset and symptoms duration, and post‐1950 generation and symptoms duration.

**FIGURE 2:**
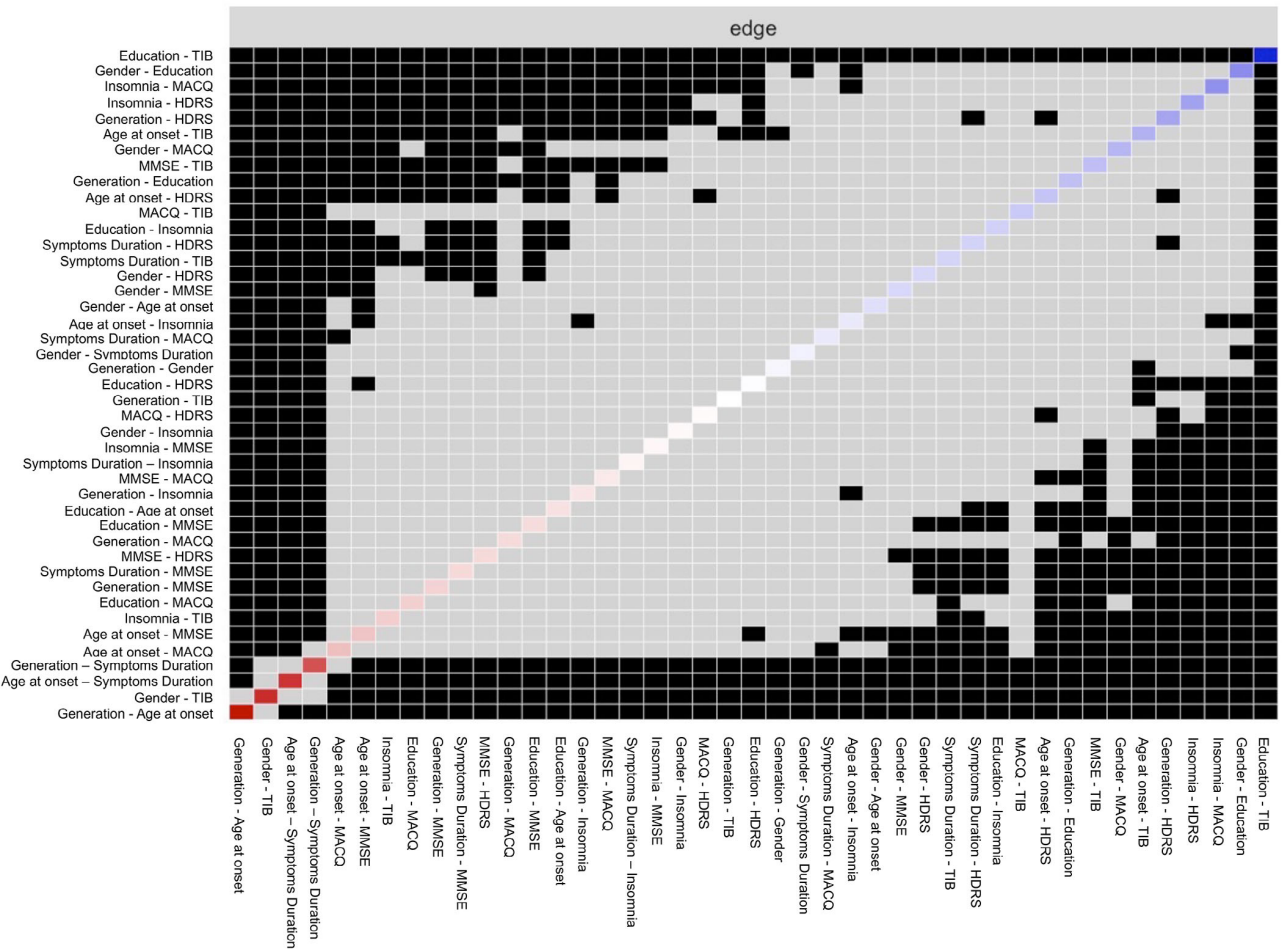
Comparison between weights of the edges in the network analysis. Black boxes indicate significant differences, gray boxes indicate non‐significant differences.

TIB scores were also directly and equally connected with age at onset, Mini‐Mental State Examination (MMSE), and MAC‐Q. Generation, age at onset was inversely connected with MAC‐Q and MMSE, with no differences among these edges. No connection was found between TIB scores and generation. Significant differences among edges are summarized in Figure 2.

**FIGURE 3:**
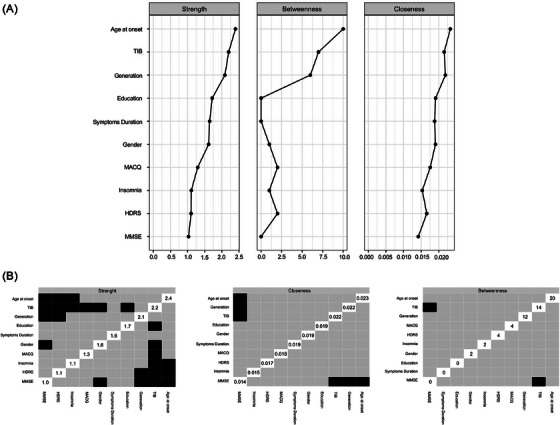
Centrality Indexes. (A) Centrality Indexes: strength, betweenness, and closeness. (B) Differences in centrality indexes among features: black boxes indicate significant differences, gray boxes indicate non‐significant differences.

Regarding centrality indexes (Figure 3), age at onset, TIB and generation had higher strength compared to MMSE and HDRS. Age at onset and TIB had higher strength than insomnia. Finally, TIB had higher strength as compared MAC‐Q, gender, and years of education. Age at onset, TIB, and generation had also a higher closeness than MMSE. Finally, TIB had a higher betweenness than MMSE.

### 3.4 Longitudinal analysis

We explored the risk of progression to MCI, considering the potential role of cognitive reserve and gender. In a follow up time > 5 years, we included 210 patients: 87 patients who progressed to MCI (SCD‐p), 123 patients who remained stable (SCD‐s). Demographic features of SCD‐p and SCD‐s are shown in eTable 2.

TIB scores were significantly lower in SCD‐p women (107.07 ± 8.08) than both SCD‐p men (116.01 ± 4.50, *p *< 0.001, d = 0.696) and SCD‐s men (115.03 ± 4.10, *p *< 0.001, d = 0.703). Moreover, SCD‐s women showed lower TIB scores than both SCD‐p men (*p *< 0.001, d = 0.609) and SCD‐s men (*p *< 0.001, d = 0.563) (Table 3).

**TABLE 3:** Demographic data in stable and progressed SCD classified according to gender.

Parameter	SCD‐s women	SCD‐p women	SCD‐s men	SCD‐p men
	*N* = 83	*N* = 63	*N* = 40	*N* = 24
Age at baseline in years	**59.51 (± 9.61)** * °	**64.24 (± 7.82)** *	60.08 (± 8.70)	**66.29 (± 7.70)** °
Age at onset in years	**55.16 (± 9.76)** ^+^, ^ç^	**59.97 (± 10.20)** ^+^, ^^^	**54.89 (± 9.33)** ^#^, ^^^	**62.25 (± 7.35)** ^#^, ^ç^
Progression time /follow‐up time	10.42 (± 4.53)	8.07 (± 5.24)	12.35 (± 6.04)	7.70 (± 5.23)
Age at progression	–	72.20 (± 8.57)	–	68.85 (± 23.30)
Generation (pre‐1950 – post‐1950)	48 ‐ 35	47 ‐ 16	29 ‐ 11	19 ‐ 5
*APOE* ɛ4+	**23.10%** ^&^	76.90%	31.60%	**68.40%** ^&^
Years of education	**12.47 (± 4.39)** ^$^	**9.74 (± 4.07)** ^$^ ^£^, ^€^	**13.05 (± 4.36)** ^£^	**14.00 (± 4.20)** ^€^
TIB	**110.39 (± 5.59)** ^α^, ^β^	**107.07 (± 8.08)** ^γ^, ^δ^	**115.03 (± 4.10)** ^α^, ^δ^	**116.01 (± 4.50)** ^β^, ^γ^
MMSE	**28.59 (± 1.86)** ^§^	**27.37 (± 2.06)** ^§^	28.42 (± 1.84)	28.10 (± 2.14)
HDRS	27.63 (± 4.69)	26.87 (± 4.00)	26.08 (± 4.36)	26.80 (± 4.64)
MAC‐Q	26.36 (± 1.96)	26.14 (± 3.47)	25.78 (± 2.87)	24.33 (± 1.65)

Values quoted in table are mean (± SD), or percentages [95% CI]. Statistically significantly different values are reported as **bold characters**. Statistical significancy after Bonferroni correction: *p *= 0.004.

Abbreviations: APOE, apolipoprotein E; HDRS, Hamilton Depression Rating Scale; MAC‐Q, Memory Assessment Clinics‐Questionnaire; MMSE, Mini‐Mental State Examination; SCD‐p, SCD patients who progressed to MCI; SCD‐s, SCD patients who remained stable with a follow up time > 5 years; TIB, *Test di intelligenza breve*.

*p = 0.003, Cohen's d = 0.289.

° *p *= 0.003, Cohen's d = 0.396.

^+^ *p *< 0.001, Cohen's d = 0.323.

^#^ *p *= 0.002, Cohen's d = 0.470.

^ *p *= 0.002, Cohen's d = 0.361.

^ç^ *p *= 0.003, Cohen's d = 0.406.

^&^ χ^2 ^= 19.32, *p *< 0.001, Cramer's V 0.533.

^$^ *p *< 0.001, Cohen's d = 0.349.

^£^ *p *< 0.001, Cohen's d = 0.424.

^€^ *p *< 0.001, Cohen's d = 0.538.

^α^ *p *< 0.001, Cohen's d = 0.563.

^β^ *p *< 0.001, Cohen's d = 0.609 .

^γ^ *p *< 0.001, Cohen's d = 0.696.

^δ^ *p *< 0.001, Cohen's d = 0.703.

^§^ *p *< 0.001, Cohen's d = 0.335.

A logistic regression analysis was performed to ascertain the effects of age at onset, TIB, gender, years of education, MMS,E and *APOE* ɛ4 on the likelihood to progress to MCI (eTable 3). The regression model was statistically significant (χ^2 ^= 36.82, *p *< 0.001). The model explained 32.9% (Nagelkerke R^2^) of the variance and correctly classified correctly 76% of cases. Sensitivity was 75%, specificity was 77%, positive predictive value was 76.19%, and negative predictive value was 76.11%. Only three were statistically significant: age at onset (B = 0.092, *p *< 0.001, OR = 1.10, 95% confidence interval [CI] 0.04:0.14), TIB (B = ‐0.124, *p *= 0.018, OR 0.88, 95% CI ‐0.23:‐0.02), and *APOE* ɛ4 (B = 1.401, *p *= 0.002, OR = 4.06, 95% CI 0.49–2.31). In more details, *APOE* ɛ4 carriers had 4.06 odds to progress to MCI than non‐carriers. Increasing age at onset was associated with increased risk of progression to MCI, while increasing TIB scores was associated with a reduction of the likelihood to progress to MCI.

We also performed a multiple regression analysis to predict time of progression to MCI considering age at onset, TIB, gender, years of education, and *APOE* ɛ4 as independent variables. The multiple regression model significantly predicted time of progression to MCI (F[5, 124] = 4.37, *p *= 0.001, adj. R^2 ^= 0.116). Among the covariates, age at onset (B = ‐0.21 [95% CI ‐0.31: ‐0.09], *p *< 0.001), TIB (B = 0.21 [95% CI 0.02: 0.40], *p *= 0.033), and years of education (B = ‐0.42 [95% CI ‐0‐72:‐0.13], *p *= 0.005) were statistically significant (eTable 4).

## 4 DISCUSSION

Our study aimed to delve into the unresolved issue of gender differences in CR in SCD, exploring possible factors underlying the disadvantage observed in women and assessing whether these gender differences impact the risk of cognitive decline progression.

SCD women showed lower premorbid intelligence (measured by TIB)^8^, ^24^; however, the cause of this difference remains unclear. Several studies have suggested that these disparities might be explained, at least in part, by social factors, as many CR contributors are highly gendered, including education, occupation, physical activity, and social support.^24^ To evaluate the potential role of social factors in explaining gender differences in CR in SCD, “generation” was used as a proxy for socio‐cultural influences on CR development. We hypothesized that women born after 1950 had greater opportunities for education and employment. Our results showed that post‐1950 women had more years of education, yet their premorbid intelligence levels were similar to those of pre‐1950 women. This counterintuitive finding may suggest a disadvantage for women in building CR, as increased education did not translate into higher TIB scores. These findings reinforce the link between female gender and CR.^25^, ^26^ However, determining what comes first is a chicken and egg situation. To disentangle this complex relationship, and to clarify the role of socio‐cultural factors, we estimate a MRF network. The primary aim of this analysis was to investigate the role of gender as a contributor of CR, independently from generation‐related socio‐cultural factors. The network revealed two major clusters: one including TIB, gender, and education; the other including age at onset, generation, and symptoms duration. The network showed a strong connection between TIB and education, since education is a contributor in CR construction.^5^ TIB and gender resulted strongly connected too, but their relation was not mediated by generation. Thus, we might hypothesize that socio‐cultural factors may not fully account for the observed gender differences in CR. The question still remains unsolved, leading to the hypothesis that other factors may underlying the relationship between female gender and CR. Previous studies had suggested that biologic and genetic factors might be implied in these gender differences.^27^, ^28^, ^29^ In particular, gender differences in CR may partly stem from the influence of sex hormones on brain function^30^, ^31^, ^32^ and of genetic factors, which influences many aspects of human brain from ion channels to brain morphology.^29^, ^33^

The lack of influence of belonging generation, thus of socio‐cultural factors, on gender differences in CR might be explained by the complexity of different occupations. Occupations traditionally classified as “manual,” such as housework, may require significant cognitive skills like planning, problem‐solving, and emotional intelligence.^24^ For example, women of the pre‐1950 generation, often “farm wives,” were responsible for finances, caregiving, and household management. Santabarbara et al. found that farming contributed more to CR than blue‐ or white‐collar jobs for both genders.^34^ Further research should investigate whether gender differences in CR in SCD are truly independent of socio‐cultural factors by examining occupation, education, and activities separately.

In the second cluster, age at onset is strongly connected with generation, indicating that post‐1950 generation patients referred self‐experienced cognitive decline earlier than pre‐1950 generation's ones. This may reflect greater public awareness of AD and brain health, and a greater likelihood of seeking medical evaluation.^2^, ^35^

A connection between TIB and age at onset was identified too, thus reflecting Stern's theory, proposing that people with higher CR levels experience symptoms later than those with lower CR.^5^, ^6^, ^8^

We also found that women reported greater severity of both cognitive complaints and depressive symptoms. This is consistent with previous studies, which suggest women are more concerned about memory loss than men.^8^, ^25^ Although depression is often associated with SCD, the higher prevalence of depressive symptoms in women merits further investigation.^26^

Consequently, we conducted a longitudinal analysis, to assess whether these gender differences in CR affect the risk of progression from SCD to MCI. Women with higher TIB were more frequently part of the SCD‐stable group, suggesting a possible advantage in copying pathology and delaying the onset of cognitive decline (or not manifesting it at all), according to Stern's model.^5^ Moreover, the logistic regression analysis showed an influence of age at onset of SCD, *APOE* ɛ4, and TIB, but not gender, on the risk of progression to MCI. Indeed, increasing premorbid intelligence was associated with a reduce likelihood of progressing to MCI. Despite the link between CR and gender, the risk of progression seems not to be influenced by gender. This point is challenging to discuss. The disadvantage of women having lower CR than men might be probably mitigated by something that levels the risk of progression between men and women. Further studies are needed to better understand which factors might have a role in mitigating the risk of progression to MCI in women despite the disadvantage in CR.

The role of age at onset and *APOE* ɛ4 genotype in progression risk is expected. SCD onset after age 60 increases the risk of objective cognitive decline, classifying it as SCD plus.^2^ In our cohort, *APOE* ɛ4 also raised the risk of progressing to MCI, confirming its known role in SCD.^3^ Prior studies have shown that *APOE* ɛ4 increases the risk of cognitive decline progression in SCD.^36^, ^37^, ^38^ Additionally, age at onset, education, and premorbid intelligence (but not gender) influence the time to MCI progression.^6^, ^39^

This study has some limitations. First, the absence of biomarker data limits insights into pathology load, which is central to the CR hypothesis. Without such data, the underlying etiology of MCI remains unclear; consequently, some cases may be due to non‐AD neurodegenerative diseases, non‐neurodegenerative conditions, or mixed pathologies. This heterogeneity may have contributed to the lack of a detectable gender effect on progression risk. Second, using generation as a proxy for socio‐cultural factors is simplistic; more detailed measures (e.g., education, occupation, leisure) are needed. Additionally, as a clinic‐based cohort, sampling bias may be present, and the lack of a control group prevents generalizing gender‐CR associations beyond SCD. Moreover, as our cohort consisted primarily of white Italian patients, our findings may not be generalized to other ethnic groups. Finally, SCD has been considered as a homogeneous group, despite recent evidence suggesting distinct subtypes with different prognoses.^40^ Nonetheless, this study has some remarkable strengths such as a relatively large and well‐characterized SCD sample, a rich dataset, and a long‐term follow‐up of over 5 years.

In conclusion, with new DMTs now available,^41^, ^42^, ^43^ early intervention in AD is more relevant than ever, though the ideal timing remains uncertain. SCD is considered a promising starting point, as brain function is still intact. However, its heterogeneity requires improved risk stratification, with CR playing a key role.^44^ While CR shows gender differences—often disadvantaging women—only CR, not gender, appears to influence progression risk. Clarifying this complex gender‐CR relationship could enhance personalized approaches and improve risk assessment in SCD.

## CONFLICT OF INTEREST STATEMENT

The authors declare that they have no competing interests. Author disclosures are available in Supporting Information.

## ETHICS APPROVAL AND CONSENT TO PARTICIPATE

Local ethics committees approved the study at each site, and all participants provided written informed consent. The study was conducted according to the Declaration of Helsinki.

## Supporting information



Supporting Information

Supporting Information

## Data Availability

All study data, including raw and analyzed data, and materials that support the findings of this study are available from the corresponding author (V.B.) upon reasonable request.
