# Correction to “Gender differences in Cognitive Reserve: An impact on progression in Subjective Cognitive Decline?”

**DOI:** 10.1002/dad2.70191

**Published:** 2025-09-21

Giulia G, Valentina M, Alice C, et al. Gender differences in cognitive reserve: An impact on progression in subjective cognitive decline? *Alzheimers Dement (Amst)*. 2025;17(3): e70174. doi:10.1002/dad2.70174

In the list of authors, the first names and surnames were inverted, leading to an incorrect attribution of the publication. In fact, the citation of the article appears as Giulia et al. instead of Giacomucci et al., due to the inversion of first name and surname.

The correct authors’ names and surnames (indicating first the given name and then the family name) are the following:

Giulia Giacomucci, Valentina Moschini, Alice Ceccarelli, Sonia Padiglioni, Carmen Morinelli, Salvatore Mazzeo, Chiara Crucitti, Giulia Galdo, Filippo Emiliani, Silvia Bagnoli, Assunta Ingannato, Elisa Marcantelli, Benedetta Nacmias, Sandro Sorbi, Valentina Bessi

We apologize for this error.

