# Supplementary material for: Gender differences in cognitive reserve: An impact on progression in subjective cognitive decline?
Source: Alzheimers Dement (Amst). 2025 Aug 26;17(3):e70174. doi: 10.1002/dad2.70174 (PMC12381356; doi:10.1002/dad2.70174)
Supplement: Supplementary file 1 — Supporting Information [file DAD2-17-e70174-s001.docx]

**Supplementary Materials**

*eTable 1. Comparison of demographic data according to generation*

|  | Pre-1950 generation | Post-1950 generation |
| --- | --- | --- |
|  | n = 249 | n = 191 |
| Age at baseline in years | **67.3 (± 6.8) *** | **56.0 (± 7.8) *** |
| Age at onset in years | **63.1 (± 7.6) ^** | **52.0 (± 9.2) ^** |
| Disease duration in years | 4.2 (± 3.5) | 4.0 (± 34.2) |
| Gender (F - M) | 164 - 85 | 140 - 51 |
| Family history of AD | 52.8% | 87.5% |
| *APOE* ɛ4+ | 29.8% | 26.3% |
| Insomnia | 56.6% | 51.8% |
| Years of education | **11.6 (± 4.6) ^§^** | **13.5 (± 43.6) ^§^** |
| TIB | 111.5 (± 7.2) | 110.6 (± 6.3) |
| MMSE | 28.1 (± 1.9) | 27.8 (± 2.2) |
| HDRS | **26.4 (± 3.9) ^+^** | **28.0 (± 4.9) ^+^** |
| MAC-Q | 25.9 (± 3.1) | 25.8 (± 3.1) |

Values quoted in table are mean (± SD), or percentages. Statistically significantly different values are reported as **bold characters**. Statistical significancy after Bonferroni correction: *p*=0.004. TIB: *Test di intelligenza breve*; HDRS: *Hamilton depression rating scale*; MAC-Q: *Memory assessment clinics-questionnaire.* * *p*<0.001, Cohen’s d=1.536; ^ *p*<0.001, Cohen’s d=1.314; ^§^ *p*<0.001, Cohen’s d=0.455; ^+^ *p*=0.002, Cohen’s d=0.372.

*eTable 2. Comparison of demographic data between SCD-p and SCD-s*

|  | SCD-p | SCD-s |
| --- | --- | --- |
|  | n = 87 | n = 123 |
| Age at baseline in years | **64.8 (± 7.8) *** | **59.7 (± 9.3) *** |
| Age at onset in years | **60.6 (± 9.5) ^** | **55.1 (± 9.6) ^** |
| Progression time /Follow up time | 12.2 (± 5.5) | 11.0 (± 5.1) |
| Age at progression | 71.3 (± 14.2) | - |
| Gender (F - M) | 63 – 24 | 83 - 40 |
| Generation (pre-1950 – post-1950) | 66 - 21 | 77 – 46 |
| *APOE* ɛ4+ | **45.8% ^+^** | **16.2% ^+^** |
| Years of education | 10.9 (± 4.5) | 12.7 (± 4.4) |
| TIB | 109.4 (± 8.3) | 111.8 (± 5.6) |
| MMSE | **27.6 (± 2.1)** **°** | **28.5 (± 1.8)** **°** |
| HDRS | 26.8 (± 4.1) | 27.1 (± 4.7) |
| MAC-Q | 25.7 (± 3.2) | 26.1 (± 2.3) |

Values quoted in table are mean (± SD), or percentages [95% C.I.]. Statistically significantly different values are reported as **bold characters**. Statistical significancy after Bonferroni correction: *p*=0.004. TIB: *Test di intelligenza breve*; HDRS: *Hamilton depression rating scale*; MAC-Q: *Memory assessment clinics-questionnaire.* * *p*<0.001, Cohen’s d=0.316; ^ *p*<0.001, Cohen’s d=0.360; ° *p*<0.001, Cohen’s d=0.166; ^+^*APOE* ɛ4+ χ^2^=15.01, *p*<0.001, Cramer’s V 0.32

*eTable 3. Logistic regression model for the risk of progression to MCI*

|  |  | **B** | ***p*** | **OR** | **95% C.I.** | |
| --- | --- | --- | --- | --- | --- | --- |
|  |  |  |  |  | **lower** | **upper** |
| Progression to MCI | Age at onset | 0.092 | **<0.001** | 1.10 | 0.04 | 0.14 |
|  | TIB | -0.124 | **0.018** | 0.88 | -0.23 | -0.02 |
|  | Gender | 0.084 | 0.870 | 1.09 | -0.92 | 1.09 |
|  | Years of education | 0.100 | 0.150 | 1.10 | -0.04 | 0.24 |
|  | MMSE | -0.178 | 0.090 | 0.84 | -0.38 | 0.03 |
|  | *APOE* ɛ4 | 1.401 | **0.002** | 4.06 | 0.49 | 2.31 |

Regression Coefficients (B), p-value (*p*), Odds Ratio (OR) and 95% Confidence Intervals (95% C.I.) for covariates included in the regression models are reported. Significant differences at *p*<0.05, **in bold characters**.

*eTable 4. Multiple regression model for time of progression to MCI*

|  | B | 95% C.I. for B | | β | *p* |
| --- | --- | --- | --- | --- | --- |
|  |  | lower | upper |  |  |
| (Constant) | 5.75 | -14.01 | 25.52 |  | 0.565 |
| Age at onset | -0.21 | -0.31 | -0.09 | -0.34 | **<0.001** |
| TIB | 0.21 | 0.02 | 0.40 | 0.26 | **0.033** |
| Gender | -1.63 | -3.97 | 0.71 | -0.28 | 0.169 |
| Years of education | -0.42 | -0.72 | -0.13 | -0.37 | **0.005** |
| *APOE* ɛ4 | -1.35 | -3.41 | 0.71 | -0.23 | 0.197 |

Unstandardized Regression Coefficients (B) and 95% Confidence Intervals (95% C.I.), standardized coefficient (β) and *p*-value (*p*), are reported (significant differences at *p*<0.05).

Supplementary Figure

1. Bootstrapped confidence interval of estimated edge-weights. The red line indicates the sample values and the grey area the bootstrapped Cis. Each horizontal lines represents one edge of the network, ordered from the highest edge-weight to the edge with the lowest edge-weights.
2. Average correlations between centrality indices of network sampled with patients dropped and the original sample. Lines indicate the means and areas the range from the 2.5th and the 97.5th quartile.
